# TRF2 Controls Telomeric Nucleosome Organization in a Cell Cycle Phase-Dependent Manner

**DOI:** 10.1371/journal.pone.0034386

**Published:** 2012-04-20

**Authors:** Alessandra Galati, Frédérique Magdinier, Valentina Colasanti, Serge Bauwens, Sébastien Pinte, Ruggero Ricordy, Marie-Josèphe Giraud-Panis, Miriam Caroline Pusch, Maria Savino, Stefano Cacchione, Eric Gilson

**Affiliations:** 1 Dipartimento di Biologia e Biotecnologie, Sapienza Università di Roma, Roma, Italy; 2 Laboratoire de Biologie Moléculaire de la cellule, Université de Lyon, CNRS UMR5239, Ecole Normale Supérieure de Lyon, Lyon, France; 3 Istituto di Biologia e Patologia Molecolari del CNR, Roma, Italy; 4 Institute for Research on Cancer and Aging in Nice (IRCAN), UMR 7284 CNRS U1081 INSERM 28 Faculté de Médecine, University of Nice, Nice, France; 5 Adolf-Butenandt-Institut, Molekularbiologie, Ludwig-Maximilians-Universität, München, Germany; 6 Department of Medical Genetics, Archet 2 Hospital, CHU of Nice, Nice, France; 7 Istituto Pasteur-Fondazione Cenci-Bolognetti, Roma, Italy; Tulane University Health Sciences Center, United States of America

## Abstract

Mammalian telomeres stabilize chromosome ends as a result of their assembly into a peculiar form of chromatin comprising a complex of non-histone proteins named shelterin. TRF2, one of the shelterin components, binds to the duplex part of telomeric DNA and is essential to fold the telomeric chromatin into a protective cap. Although most of the human telomeric DNA is organized into tightly spaced nucleosomes, their role in telomere protection and how they interplay with telomere-specific factors in telomere organization is still unclear. In this study we investigated whether TRF2 can regulate nucleosome assembly at telomeres.

By means of chromatin immunoprecipitation (ChIP) and Micrococcal Nuclease (MNase) mapping assay, we found that the density of telomeric nucleosomes in human cells was inversely proportional to the dosage of TRF2 at telomeres. This effect was not observed in the G1 phase of the cell cycle but appeared coincident of late or post-replicative events. Moreover, we showed that TRF2 overexpression altered nucleosome spacing at telomeres increasing internucleosomal distance. By means of an *in vitro* nucleosome assembly system containing purified histones and remodeling factors, we reproduced the short nucleosome spacing found in telomeric chromatin. Importantly, when *in vitro* assembly was performed in the presence of purified TRF2, nucleosome spacing on a telomeric DNA template increased, in agreement with *in vivo* MNase mapping.

Our results demonstrate that TRF2 negatively regulates the number of nucleosomes at human telomeres by a cell cycle-dependent mechanism that alters internucleosomal distance. These findings raise the intriguing possibility that telomere protection is mediated, at least in part, by the TRF2-dependent regulation of nucleosome organization.

## Introduction

Telomeres are an important genome-stabilizing component of linear chromosomes. Variations in telomere status critically affect cell senescence, stem cell biology, and the development of many diseases, including cancer [1]. The importance of telomeres in governing cell fate is likely attributable to their numerous functions; they protect chromosome ends from DNA damage checkpoint machinery and repair, control the terminal replication of chromosomal DNA, localize chromosome ends within the nuclear space, and regulate gene expression [2], [3].

In most organisms, telomeres are composed of short, tandemly repeated DNA sequences, ending in a G-rich single-stranded 3′ tail. They are transcribed in a G-rich RNA named TERRA [4], which is thought to play relevant functions at telomeres. The chromatin structure of telomeres is unusual, forming a so-called telosome [5], [6]. Telosomes are essential for the preservation of chromosome stability; they control telomere length, recombination, and DNA damage checkpoints. The yeast telosome is a non-nucleosomal chromatin structure containing the telomeric DNA-binding protein Rap1p [7]. A key component of human telosomes is the shelterin complex [8], [9], a structure composed of six polypeptides (TRF1, TRF2, RAP1, Tin2, TPP1, and Pot1). Three of the shelterin components recognize directly telomeric DNA; TRF1 and TRF2 bind telomeric DNA duplexes, while Pot1 binds single-stranded 3′ overhangs. In contrast to yeast telomeric organization, the shelterin complex appears to co-localize with nucleosomes. In higher eukaryotes, most telomeric DNA is organized into tightly spaced nucleosomes [10]–[13]; moreover, a 30-nm telomeric fibre has been observed in the telomeres of chicken erythrocytes and of quiescent mouse lymphocytes [14]. Mammalian telomeric chromatin exhibits characteristics of heterochromatin [15] and triggers telomere position effects [16], [17]. Other links between telomeres and chromatin include the ATRX-dependent enrichment of the histone variant H3.3 at telomeres [18], [19] and the phosphorylation of the H2AX histone triggered by dysfunctional telomeres [20]. Furthermore, telomere shortening negatively affects histone synthesis, probably via damage signal induction [21]. Overall, it appears that the particular nature of telomeric chromatin plays a role in telomere capping, telomere length regulation, and long-range gene expression.

However, an unresolved question regarding the organization of mammalian telosomes is whether nucleosomes and components of the shelterin complex occupy different portions of the telomere, or whether they co-localize and cooperate to establish a protective telomere structure [22]. Indeed, the repeated nature of the telomeric DNA sequence suggests that the tight spacing of telomeric nucleosomes revealed by micrococcal nuclease (MNase) digestion could reflect the organization of only a portion of the telomere. One must also consider that if nucleosomes were uniformly spaced along the entire telomere, the binding of TRF1 or TRF2 would be limited to the short linker DNA or to nucleosomal binding sites. Thus, whether specific telomeric proteins compete with histone octamers for binding to telomeric sequences or cooperate to form a telomeric protective structure is still not well understood. Recent studies produced seemingly contradictory results. Overexpression of TRF2 in primary keratinocytes derived from transgenic mice increases histone spacing [23], while a loss of TRF2 expression in mouse embryonic fibroblasts did not alter nucleosome positioning [24]. This discrepancy might be due to differences in the cell systems utilized, since the effects of modulating TRF2 expression on telomere length and stability can be highly cell line-dependent [25], [26]. For example, the nucleosome reduction observed upon TRF2 overexpression [23] could be a consequence of the extremely short telomeres characteristic of the used cells [27] or of the long-term effects of TRF2 overexpression.

Here, we examined the short-term effects of increasing or decreasing TRF2 levels on nucleosome organization at human telomeres. We found an inverse correlation between the dosage of TRF2 in telomeres and the density of nucleosomes in a telomere-length independent manner. Moreover, we found that the TRF2 influence on chromatin organization is regulated during the cell cycle and can be recapitulated *in vitro*.

## Results

### TRF2 overexpression reduces nucleosome density and increases internucleosomal distance at telomeres

**Figure 1:**
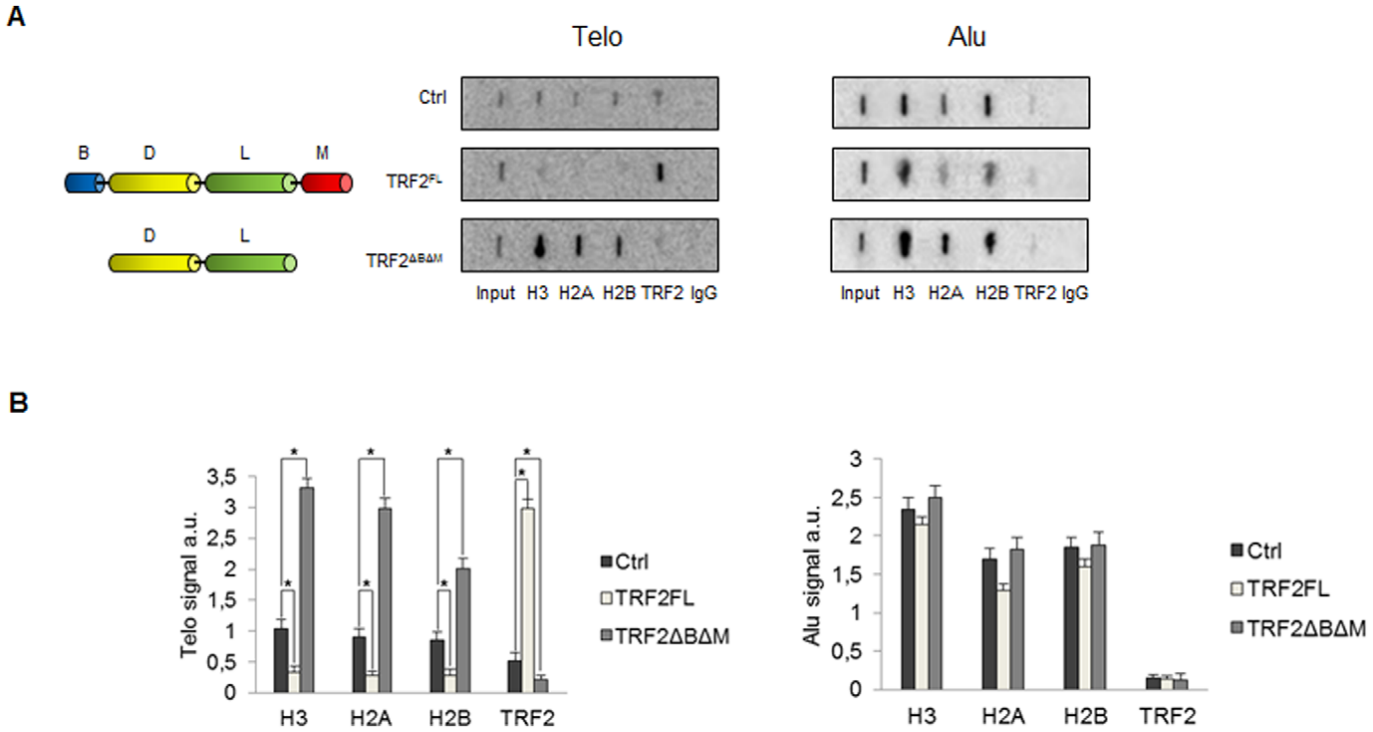
Nucleosome density at human telomeres depends on TRF2 expression. (**A**) ChIP of C33A cells overexpressing TRF2^FL^ or TRF2^ΔBΔM^ and control C33A cells using the indicated antibodies. Slot-blots were hybridized with a labelled Telo repeat probe and an Alu probe. (**B**) Quantification of the data in (**A**) expressed as probe/input hybridization signals. Error bars are s.d. of three independent experiments. Asterisks, p<0.05 based on unpaired Student's t-test.

To investigate the interplay between TRF2 and telomeric chromatin, we infected human cancer cell (C33A from cervix) with lentiviral vectors encoding TRF2 full-length or a truncated form which lacked both the N-terminal basic domain and the telobox Myb-like C-terminal DNA-binding domain (TRF2^ΔBΔM^); this mutant has a dominant negative activity, since it forms dimers with TRF2 that have a reduced ability to bind telomeres [25]. In order to observe the effect of TRF2 overexpression or depletion on histone density at telomeres, we performed chromatin immunoprecipitation (ChIP) assays 72 h post-infection. Figure 1A shows a slot-blot hybridization using telomeric (Telo) and Alu repeat (Alu) DNA probes. As previously shown, the association of TRF2 with telomeres was increased in cells overexpressing the full-length protein (TRF2^FL^) compared to control cells (infected with an empty vector), whereas the association was decreased in cells expressing TRF2^ΔBΔM^ [25]. The quantities of H3, H2A, and H2B at telomeres were inversely related to the amount of telomere-bound TRF2. When the filters were hybridized with an Alu probe, the H3, H2A, and H2B signals were not modified by TRF2 expression (Figure 1B). These experiments confirm and extend previous observations made upon long term overexpression of TRF2 in mouse cells [23], indicating that TRF2 dosage alters nucleosomal organization. The quantities of TRF2 and immunoprecipitated H3 at telomeres also varied inversely in HT1080 fibrosarcoma cells in a telomere length independent manner (see Figure S1), demonstrating that the effect of TRF2 on nucleosome organization occurs in multiple cell types. Importantly, in contrast to the study in mouse cells [23], the overexpression of full-length TRF2 did not induce a change in telomere length at the time point selected for cross-linking in our ChIP experiments (Figure S2), showing that the influence of TRF2 on nucleosome organization does not result from telomere shortening. However, we cannot rule out that at least part of the increase of histone density at telomeres upon TRF2^ΔBΔM^ expression was related to an increase of DNA damage at telomeres. As previously reported [28], Figure S2 also shows that the expression of TRF2^ΔBΔM^ led to a slight telomere lengthening in C33A cells.

**Figure 2:**
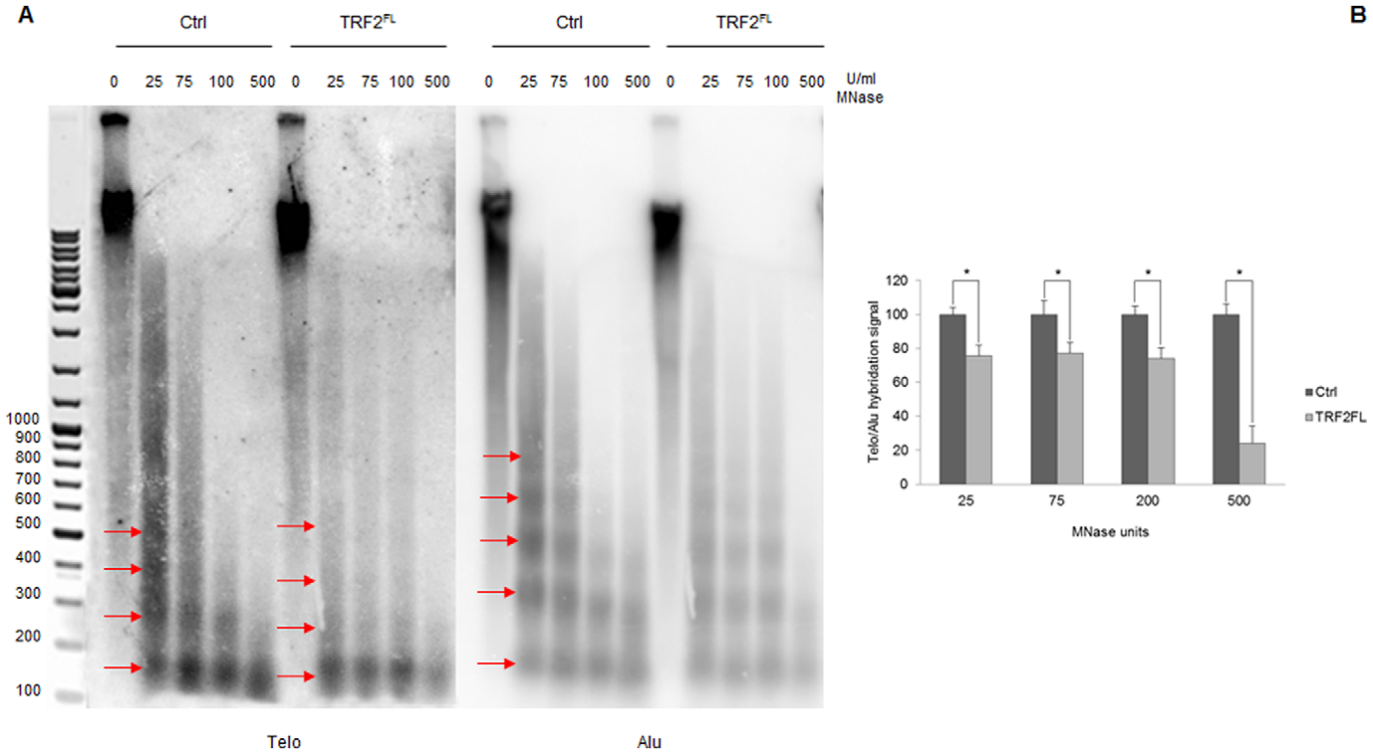
Altered nucleosome spacing at C33A telomeres. (**A**) Digestion of chromatin from C33A cells infected with an empty vector and C33A cells overexpressing TRF2^FL^ with increasing amounts of MNase. From the left: MNase digests separated on 1.5% agarose gel detected by hybridization with Telo probe; detection of telomeric nucleosomes after hybridization with Alu probe. (**B**) Ratio of the overall hybridization signal of the telomeric probe with respect to the Alu probe. Ratio values for control C33A cells have been normalized to 100. Error bars are s.d. of three independent experiments. Asterisks, p<0.05 based on unpaired Student's t-test.

In order to analyze the effect of TRF2 on the spacing of telomeric nucleosomes, we digested nuclei from control cells and C33A cells overexpressing TRF2 with micrococcal nuclease (MNase). Figure 2A reports nucleosomal ladders obtained by MNase digestion hybridized with a telomeric probe (left part) and an Alu probe (right part). Consistent with previous publications [10]–[12], MNase digestion showed that telomeric nucleosomes have a repeat size shorter than Alu nucleosomes (about 160 bp versus 180 bp for bulk nucleosomes hybridized with the Alu DNA probe; Figure 2A). Upon TRF2 overexpression bands in telomeric nucleosomal ladders appear more diffuse, indicating a less regular nucleosome spacing at telomeres. This could derive from an increase in nucleosome spacing caused by TRF2 overexpression, evident for fragments greater than a trinucleosome (Figure 2A). Our data suggest that the reduced immunoprecipitation of histones upon TRF2 overexpression might result, at least in part, from an increased internucleosomal distance. Further support to this interpretation comes from the quantification of the overall hybridization signal; we measured the radioactivity signal for every single lane and calculated the ratios of the Telo probe signals to the corresponding Alu probe signals. Figure 2B shows the ratio values in the case of C33A cells infected both with the empty vector and with TRF2^FL^. Telo/Alu ratios have been normalized to 100 in the case of control cells. Ratio values are significantly lower in TRF2 overexpressing cells (Figure 2B), indicating a higher sensitivity to MNase of telomeric chromatin upon TRF2 overexpression that could derive from lower nucleosome density. It is worth noting that decreases of the Telo/Alu ratios in TRF2^FL^ overexpressing cells are similar (about 75% of the control signal) except for the samples digested with 500 U/ml MNase (about 25% of the control signal). This could reflect the hypersensitivity to MNase of telomeric mononucleosomes [11], the main product of the digestion at this MNase concentration. These results indicate that the TRF2-mediated alterations of nucleosome organization observed by ChIP cannot be merely attributed to accessibility problems or ChIP artefacts, suggesting that TRF2 binding affects nucleosome density.

Overall, ChIP and MNase chromatin analyses revealed that TRF2 exhibits cell line-independent nucleosome reorganization properties.

### Cell-cycle regulation of TRF2-mediated telomeric remodelling

**Figure 3:**
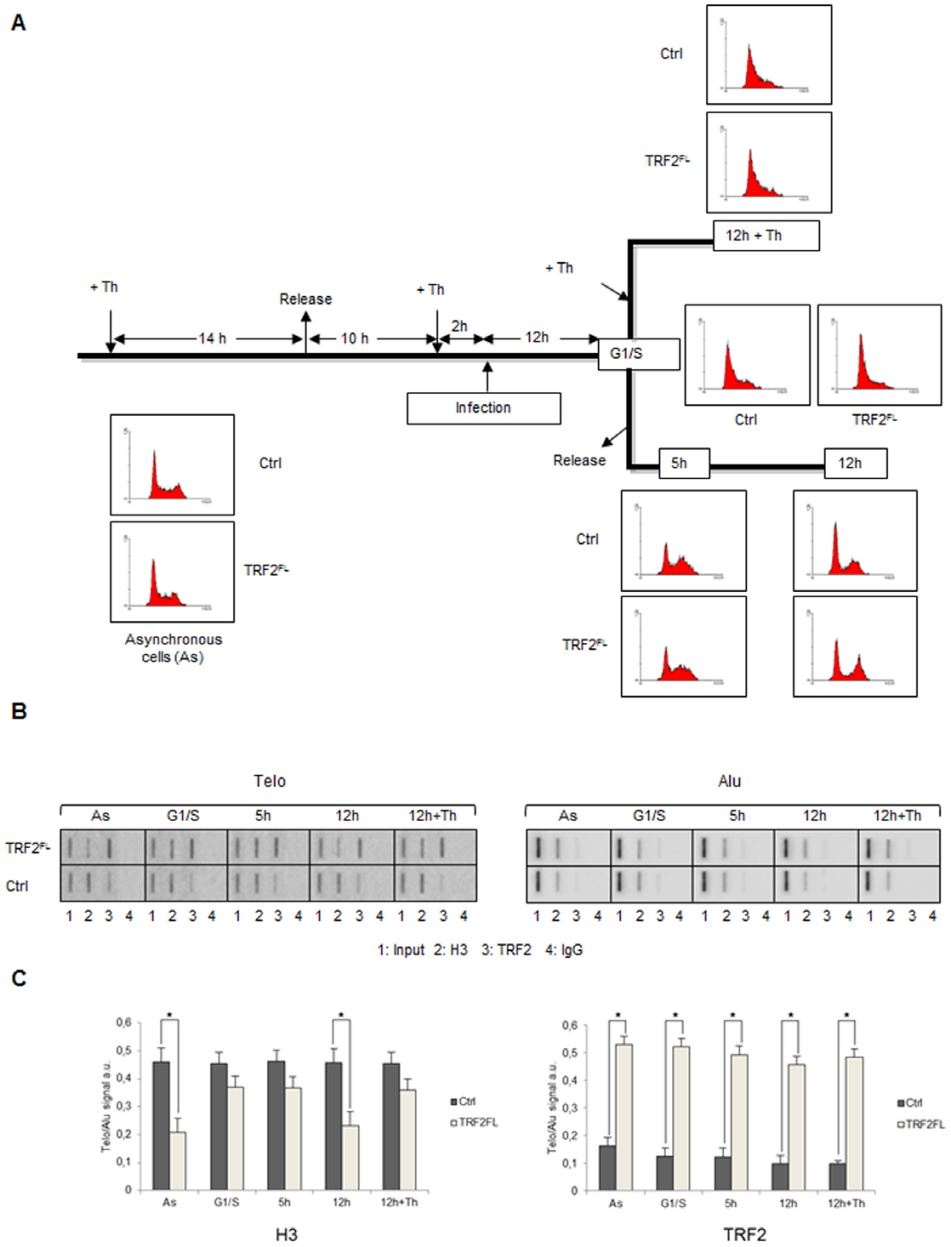
Cell-cycle regulation of TRF2-mediated telomeric remodelling. (**A**) Scheme of the synchronization experiment (see Materials and Methods for details). (**B**) ChIP of C33A cells overexpressing TRF2^FL^ and control C33A cells using the indicated antibodies and hybridized with Telo and Alu probes. From the left: slot-blots in asynchronous control cells; at the G1/S boundary; 5 hours after release from thymidine block; 12 hours after release; cells maintained in thymidine block for 12 additional hours (12 h+Th). (**C**) Quantifications of the data in (**B**) expressed as Telo/Alu hybridization signals. Error bars are s.d. of three independent experiments. Asterisks, p<0.05 based on unpaired Student's t-test.

The duplication of eukaryotic DNA requires the transient disruption of parental nucleosomes to allow progression of the replication fork [29]. TRF2-dependent variations in histone density at telomeres may therefore be influenced by nucleosome dynamics that occurs during DNA replication. To address this possibility, we synchronized C33A cells overexpressing TRF2 at the G1/S boundary by a double-thymidine treatment followed by a release in fresh medium (Figure 3A). The cell cycle distribution was examined by FACS analysis (Figure 3A). Viral infections were performed 12 h before release. The binding of histone H3 and TRF2 to telomeres was analyzed by ChIP (Figure 3B). In TRF2-overexpressing cells, TRF2 levels were increased compared to control cells at all stages of the cell cycle (G1/S, 5 h and 12 h). This is in agreement with the rapid exchange of at least a subset of TRF2 at telomeres [30]. Concomitantly, H3 levels were decreased both in asynchronous control cells and in synchronized cells 12 h post-release (i.e., at the end or shortly after S phase; Figure 3). Importantly, in cells blocked at G1/S for twelve additional hours (12+Th, Figure 3A), the incorporation of TRF2 did not lead to a significant histone H3 displacement (Figure 3C). These results show that the changes in nucleosome organization triggered by TRF2 require both telomeric DNA binding and a cell cycle-regulated event occurring between the end of the S and G1 phases.

### TRF2 affects nucleosome organization at telomeric sequences *in vitro*

We next examined the ability of TRF2 to interfere with nucleosome assembly *in vitro*. It was shown previously that the reconstitution of nucleosomal arrays by salt dilution at high saturation levels of histone octamers allows the reproduction of the tightly spaced organization of telomeric chromatin found *in vivo*, due to the absence of nucleosome positioning signals on telomeric DNA [31], [32]. Furthermore, addition of TRF2 to reconstituted telomeric nucleosomal arrays induces chromatin compaction [33], [34]. However, reconstitution by salt dilution does not allow evaluating whether TRF2 competes with nucleosome assembly, since nucleosome formation occurs at non-physiological ionic strengths that disfavour TRF2 binding. We therefore decided to use *Drosophila* embryonic extracts to assemble telomeric nucleosomal arrays [35]. This method offers several advantages, one of which being that assembly occurs in the presence of histone chaperones and ATP-dependent remodelling complexes. Furthermore, it is performed at physiological ionic strengths and allows the production of regularly spaced nucleosomal arrays [35]. In addition, since *Drosophila* has no endogenous TRF2, purified TRF2 can be added to *Drosophila* embryonic extracts to evaluate the impact of the protein on nucleosome assembly.

**Figure 4:**
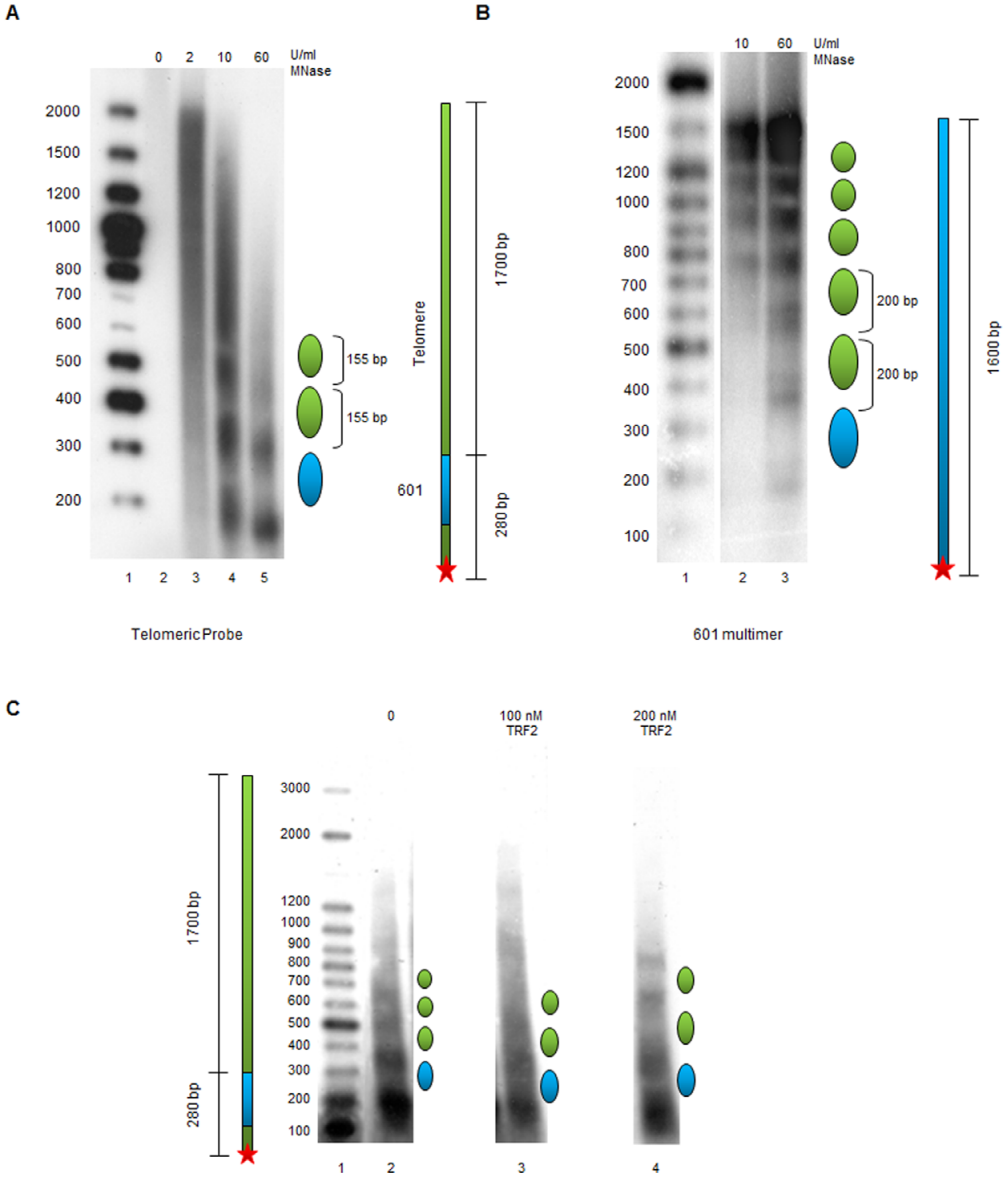
TRF2-regulated *in vitro* chromatin assembly on human telomeric DNA. (**A**) MNase digestion of chromatin assembled on the 601/telomere DNA fragment. Lane 1, labelled 100 bp DNA ladder; lane 2 to 5, assembled chromatin digested respectively with 0, 2, 10, 60 U/ml of MNase. A schematic drawing of the DNA fragment and of the nucleosomal positioning and spacing is represented on the right. (**B**) MNase digestion of chromatin assembled on the 601-200_8_ DNA fragment. Lane 1, labelled 100 bp DNA ladder; lane 2 to 3, assembled chromatin digested with MNase. (**C**) Chromatin assembly in the absence and in the presence of TRF2. Lane 1, labelled 100 bp DNA ladder; lane 2, assembled chromatin digested with 10 U/ml of MNase; lane 3, chromatin assembled in the presence of 100 nM TRF2 digested with 10 U/ml of MNase; lane 4, chromatin assembled in the presence of 200 nM TRF2 digested with 10 U/ml of MNase.

In our *in vitro* nucleosome assembly experiments, we used a construct in which the 601 DNA sequence was placed directly upstream of a 1.7-kbp human telomeric sequence. The 601 DNA is a 147 bp DNA with the highest affinity for the histone octamer known to date [36]. Since telomeric nucleosomes occupy multiple positions [37] and spontaneously slide along DNA [38], the presence of a well-positioned nucleosome represents a precise starting point to map nucleosome organization on telomeric sequences. The 601/telomere DNA construct was terminally labelled upstream of the 601 DNA sequence and the nucleosomal array assembled *in vitro* using a *Drosophila* embryonic extract. Nucleosome positioning relative to the end of the fragment was monitored by digestion with MNase and separation of the resultant DNA fragments on an agarose gel. A nucleosomal assembly reaction onto the 601/telomere DNA construct is shown in Figure 4A. Digestion of the assembled nucleosomal arrays with MNase yielded a nucleosomal ladder with a spacing of approximately 155 bp, consistent with the nucleosome spacing found *in vivo* at telomeres [10]–[12]. In comparison, MNase digestion of a nucleosomal array assembled on a 1,600-bp DNA fragment containing eight tandem 200-bp repeats of the 601 DNA yielded a regular 200 bp nucleosome spacing (Figure 4B). These data strongly suggest that the tight nucleosome spacing found at telomeres is a sequence-dependent phenomenon specific to telomeric chromatin. To further exclude the possibility that the short telomeric nucleosome spacing observed is an artefact of our experimental conditions, we used the *Drosophila* extracts to form nucleosomal arrays on a non-repetitive DNA sequence, namely a linearized pUC18 plasmid containing only one 601 repeat at the end of the fragment (Figure S3). Also in this case MNase digestion revealed a nucleosome spacing of about 200 bp.

Next, we analyzed whether TRF2 could affect nucleosome assembly on telomeric DNA. When nucleosomal arrays were assembled in the presence of TRF2, the distance between telomeric nucleosomes increased and became less regular (Figure 4C). In particular, the increase in spacing seems directly related with nucleosome distance from the 601 sequence. The addition of a non-specific protein such as bovin serum albumin does not modify telomeric internucleosomal distances (Fig. S4), supporting the hypothesis that the increase in nucleosome spacing is due to the specific binding of TRF2 to telomeric DNA and not to non-specific effects. The *in vitro* chromatin assembly data are consistent with the results of MNase digestion analyses performed on cells overexpressing TRF2 (Figure 2) [23], and support a highly dynamic view of human telomeric chromatin.

## Discussion

This work demonstrates that TRF2 is capable to modify nucleosome organization both *in vitro* and *in vivo*. Chromatin immunoprecipitation experiments reveal an inverse correlation between TRF2 dosage and nucleosomes at telomeres of cancer cells (Figure 1). The number of detected telomeric nucleosomes increases in TRF2-depleted cells, while less telomeric nucleosomes are observed in TRF2-overexpressing cells. These data suggest that TRF2 reduces either the accessibility to anti-histone antibodies or the amount of nucleosomes or both. However, in TRF2 overexpressing cells nucleosome spacing at telomeres increases and the telomeric chromatin appears more accessible to MNase (Figure 2); this data argue against a model where TRF2 reduces the accessibility of telomeric chromatin favouring the alternative possibility, i.e. that TRF2 reduces nucleosome density at telomeres. These results are in agreement with previous results in mice showing that TRF2 overexpression decreases the levels of histones H3 and H4 and increases nucleosome spacing at telomeres [23]. In addition, the current study reveals several new key points. First, nucleosome reorganization is a short-term effect of TRF2 binding and does not correlate with changes in telomere length. We further showed that the incorporation of TRF2 at telomeres in G1/S cells does not trigger nucleosome reorganization, while passage through S/G2/M does. Importantly, the ability of TRF2 to alter nucleosome organization is supported by *in vitro* chromatin assembly experiments using *Drosophila* embryonic extracts that are devoid of shelterin components. In the absence of TRF2, the distance between telomeric nucleosomes reproduces the short spacing found *in vivo* [10], [11], suggesting that this could be an intrinsic characteristic of telomeric sequences. When TRF2 is added during the assembly, nucleosome spacing increases and becomes irregular, indicating that TRF2 possesses the intrinsic ability to change nucleosome organization.

**Figure 5:**
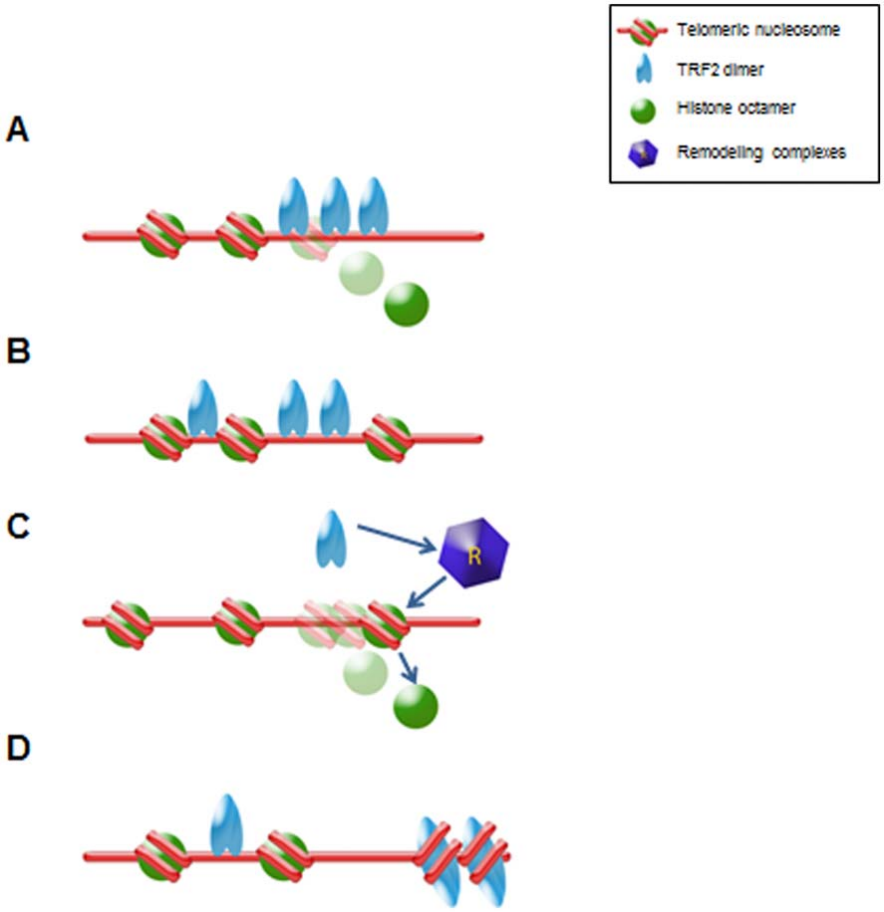
Models for TRF2-induced remodeling of telomeric chromatin. See the text for details.

Several non-exclusive mechanisms could explain how TRF2 reduces nucleosome density at telomeres. First, TRF2 binding could displace histone octamers (Figure 5A). However, this hypothesis appears unlikely since: i) the incorporation of TRF2 at telomeres in G1/S cells do not trigger apparent nucleosome displacement; ii) both TRF1 and TRF2 are unable to dissociate nucleosomes *in vitro*, even at a high protein/nucleosome ratio [39] (A. Galati, M. Savino, S. Cacchione, unpublished data), despite the fact that telomeric nucleosomes are less stable than bulk nucleosomes [40]; addition of TRF2 to reconstituted telomeric nucleosome arrays causes chromatin compaction but no apparent dissociation of histones [33] iii) TRF2 has a very low affinity for nucleosomal telomeric binding sites (A. Galati, M. Savino, S. Cacchione, unpublished data), which makes TRF2 different from other telomeric proteins such as hTRF1 [39] and yeast Rap1 [41]. Second, TRF2 may bind between nucleosomes, interacting with nucleosome borders, thereby increasing their spacing (Figure 5B). This may result from an enhancement of the intrinsic mobility of telomeric nucleosomes [38]. This mechanism has been recently proposed to explain TRF1-induced nucleosome mobility *in vitro* [42]. Third, TRF2 could act by recruiting ATP-dependent remodeling complexes to telomeres that could mediate nucleosome sliding and/or disruption (Figure 5C). Fourth, TRF2 could compete with histones for DNA binding during nucleosome assembly (Figure 5D). In favour of this hypothesis, TRF2 wraps DNA around itself in a right-handed orientation [43]–[45]. This could disfavour nucleosome formation at or in the close proximity of TRF2-DNA complexes by imposing torsional constrains.

The TRF2-mediated chromatin changes will undoubtedly deserve further investigation. Considering that altered TRF2 expression levels result in telomere dysfunction and instability, a tempting hypothesis is that TRF2 regulation of nucleosome density is an important factor in the establishment of a protective telomere structure. It is worth noting that another telomeric protein, yeast Rap1, forms a nucleosome-free region at telomeres [5]; interestingly, in the case of yeast Rap1, the effect on chromatin organization can also be observed outside telomeres: nucleosome occupancy at several yeast promoters containing Rap1 sites was also shown to be reduced [46], [47]. Thus, the chromatin modulation controlled by TRF2 might also be involved in the regulation of expression of a network of genes located throughout the genome [48], [49]. This would provide a mechanistic link between a telomere's functional state and cellular transcriptional programs.

## Materials and Methods

### Cell culture and synchronization

Human cell lines C33A (carcinoma, cervix), human fibrosarcoma HT1080 and kidney cell line 293 T were maintained in Dulbecco's modified Eagle's medium (DMEM) supplemented with 10% fetal calf serum (FCS) and 100 U/ml penicillin plus 100 mg/ml streptomycin.

To synchronize the human C33A cells at the G1/S boundary, double-thymidine block was performed. Cells were arrested with 2 mM thymidine for 14 h, and then released to fresh medium for 10 h followed by second treatment of 2 mM thymidine for 14 h.

### Lentivirus production

Lentivirus for the transfection was prepared essentially as described [50]. 8,6 µg of VSVg-pseudotyped self inactivating (SIN) lentiviruses, expressing empty vector, TRF2^FL^, or TRF2^ΔBΔM^, 8.6 µg of Lenti-Delta 8.91 and 2.8 µg of VSV-g were introduced into 5×10^6^ T293 cells in DMEM, supplemented with 10% Fetal Calf Serum at 37° in 5% CO_2_ in 10 cm dish, through calcium phosphate mediated transfection. Virus containing supernatants were collected after 48 and 72 hours, passed through a 0.45 µm Millipore filter and used to infect C33A and HT1080 cells. The efficiency of infection was determined by flow cytometry analysis of GFP expression.

### Cell cycle analysis

DNA content and bromodeoxyuridine (BrdU) incorporation were determined by flow cytometry (Coulter Epix XL). Briefly, 30 min before harvesting, 45 µM BrdU was added to the culture medium. Cells were then fixed in a 1∶1 methanol: PBS mixture and DNA was denaturated in 3 N HCl for 60 min. After neutralizing in 0.1 M sodium tetraborate, cells were incubated with a mouse monoclonal anti-BrdU antibody (Becton & Dickinson) for 1 h at room temperature, washed in PBS+0.5% Tween-20 and then incubated for 45 min with Alexa Fluor 488-conjugated anti mouse antibody (Molecular Probes, Eugene, OR). After extensive washing, cells were resuspended in PBS containing 10 µg/ml propidium iodide and analysed for their DNA content (red fluorescence) and BrdU incorporation (green fluorescence). Ten thousand events were collected for each sample. DNA histograms and biparametric dot plots of DNA and BrdU content were obtained using WinMDI software.

### Chromatin immunoprecipitation assays (ChIP)

For ChIPs, 3×10^6^ cells were fixed in 1% formaldehyde for 15 min at RT on a shaking platform. Cross-linked cells were washed twice with cold PBS, scraped and lysed at a density of 20×10^6^ cells/ml for 10 min at 4°C in 1% SDS, 50 mM Tris-HCl (pH 8.0) and 10 mM EDTA. Lysates were sonicated to obtain chromatin fragments <1 kb and centrifuged for 15 min in a microfuge at RT. Chromatin was diluted 1∶10 with 1.1% Triton-X100, 2 mM EDTA, 150 mM NaCl and 20 mM Tris-HCl (pH 8.0) and precleared with a 50% salmon sperm DNA/protein A agarose slurry (Millipore). Chromatin fragments were incubated with one of the following antibodies at 4°C overnight on a rotating platform: 5 µg of anti-H2A (Abcam), anti-H2B (Abcam), rabbit polyclonal anti-Histone H3 (Millipore), 10 µg of monoclonal anti-TRF2 (Imgenex), and 1 µg IgG (SIGMA). Salmon sperm DNA/protein Agarose beads (60 µl) were then added and the incubation continued for 1 hour. Immunoprecipitated pellets were washed with 0.1% SDS, 1% Triton-X100, 2 mM EDTA, 20 mM Tris-HCl (pH8.0) and 150 mM NaCl (one wash); 0.1% SDS, 1% Triton-X100, 2 mM EDTA, 20 mM Tris-HCl (pH 8.0) and 500 mM NaCl (one wash); 0.25 M LiCl, 1% Nonidet P-40, 1% sodium deoxycholate, 1 mM EDTA and 10 mM Tris-HCl, pH 8.0 (one wash); and 10 mM Tris-HCl (pH 8.0) and 1 mM EDTA (two washes). Chromatin was eluted from the beads with 250 µl 1% SDS and 0.1 M NaHCO_3_. After adding 20 µl of 5 M NaCl, crosslinks were reversed for 4 h at 65°C. Samples were supplemented with 20 µl of 1 M Tris-HCl (pH 6.5), 10 µl of 0.5 M EDTA, 20 µg of RNase A and 40 µg of proteinase K and incubated for 1 h at 45°C. DNA was then recovered by phenol/chloroform extraction and ethanol precipitation, slot-blotted onto a Hybond N+ membrane and hybridized with a telomeric probe or Alu probe labeled by random priming. Filters were scanned with Typhoon 9200 phosphoimager (GE Healthcare). The quantification of the signal was done using the ImageQuant software. For total DNA samples, aliquots corresponding to 1/10 dilution of the amount of lysate used in the immunoprecipitation were processed along with the rest of the samples at the step of reversing the crosslinks. We calculated the amount of telomeric or centromeric DNA immunoprecipitated in each ChIP using the ratio between the immunoprecipitated fraction and the corresponding total DNA sample (Input).

### Telomere length measurements

For TRF determination, 15 µg of DNA were digested with restriction enzymes Hinf I (10 U) and Rsa I (10 U; Roche), and electrophoresed on 0.8% agarose gel. Then, DNA was denatured, neutralized, transferred to a nylon membrane (Hybond N, Amersham International, Buckinghamshire, UK) and cross-linked with ultraviolet light. The membrane was hybridized with 50-end [γ-32P]deoxyadenosine triphosphate labeled telomeric oligonucleotide probe (TTAGGG)_4_ at 42°C for 2 h in a rapid hybridization buffer (QuikHyb Hybridization Solution, Stratagene, La Jolla, USA). After washing, the filters were autoradiographed (Hyperfilm-MP; Amersham) with an intensifying screen at −80°C for 24 h and the autoradiographs were scanned and the mean telomere length calculated.

### Micrococcal nuclease digestion of nuclei

Nuclei were isolated and digested with micrococcal nuclease (MNase) as described [11]. Briefly, cells were trypsinized, suspended in growth medium, and harvested by centrifugation at 1,500 rpm for 5 min. Cells were suspended at 2×10^6^ cells/ml in buffer A (100 mM KCl, 10 mM Tris [pH 7.5], 3 mM MgCl2, 1 mM CaCl2, 0.5 mM PMSF), washed twice with buffer A, then resuspended at 5×10^6^ cells/ml in buffer A with 0.6% Nonidet P-40 to lyse cells. After gentle mixing and incubation on ice for 5 min, nuclei were harvested at 2,000 rpm for 5 min and resuspended in buffer A without NP-40 at 2.5×10^7^ cells/ml. Cells were homogenized in a dounce homogenizer using a tight B-type pestle. Aliquots of 150 µl were digested for 5 min at 30°C with MNase (Worthington) at concentrations ranging from 25 to 500 U/ml. Reactions were stopped by adding one volume of TEES/proteinase K (10 mM Tris HCl [pH 7.5], 10 mM EDTA, 10 mM EGTA, 1% SDS, 50 µg/ml proteinase K) and incubated at 37°C from 2 hours to overnight. DNA was extracted with phenol/chloroform, precipitated with isopropanol in the presence of 0.2 M sodium acetate (pH 5.5), and resuspended in 500 µL TE (10 mM Tris, 1 mM EDTA, pH 8.0). Samples were run on 1.5% agarose gels, transferred to a nylon membrane and hybridized sequentially with a telomeric probe and with an Alu probe. Filters were scanned with Typhoon 9200 phosphoimager (GE Healthcare) and images analyzed with Imagequant software and corrected using median local background subtraction.

### Plasmids

The plasmid pUC18/601-200_8_ was a kind gift from D. Rhodes. To obtain the 601/Telomere construct, a 601 monomer was extracted from the pUC18/601-200_8_ plasmid by cutting with the AvaI restriction enzyme. The DNA fragment was then ligated to two adapters containing the BamHI restriction site (Adapter I: 5′-GCCGATGGATCCTATGTCAC-3′, 5′-CCGAGTGACATAGGATCCATCGGC-3′; Adapter II: 5′-TCGGGTTCAAGGGATCCGCATCC-3′, 5′-GGATGCGGATCCCTTGAAC-3′). The construct was digested with BamHI and inserted in the BamHI site of the pCMV-Telo plasmid [17] upstream of the 1700 bp of TTAGGG repeats.

### Protein purification

6xHis -tagged TRF2 was expressed in BL21(D3) cells and purified as previously described [43]. Briefly, supernatant from a cell lysate was bound to Ni-NTA slurry (Qiagen). After several washes with Wash Buffer (50 mM Hepes [pH8], 10 mM β-mercaptoethanol, 500 mM KCl, 20 mM Imidazole, 1 mM PMSF, 10% Glycerol), the protein was eluted with the same buffer containing 300 mM Imidazole and dialyzed against Wash Buffer. Protein concentration was assessed by Bradford assay (SIGMA). A SDS-PAGE gel of the purified protein is shown in Figure S5.

### Chromatin assembly and micrococcal nuclease analysis

The pCMV-601Telo plasmid was digested with Alw44I and the resulting 4400 bp DNA fragment was gel purified and biotinylated by filling in with klenow enzyme and biotin-11-dUTP (Fermentas). The biotinylated fragment was then digested with Bsp1407I to generate a fragment of about 2000 bp, which was gel-purified and terminally labelled by filling in with klenow enzyme and [α-^32^P]dATP. The 601-200_8_ DNA sequence was prepared by digesting the pUC18/601-200_8_ plasmid with XbaI and biotinylated as described above. The biotinylated plasmid was then digested with EcoRI, gel-purified and labeled by filling in with the klenow enzyme and [α-^32^P]dATP.

For the assembly reaction 1 µg of labelled DNA was bound to Dynabeads streptavidin M-280 (Dynal) and then assembled with *Drosophila* embryo extracts essentially as described [35]. To the DNA, we added 40 µl embryo extract, 40 µl EX buffer (10 mM Hepes-KOH (pH 7.6), 1.5 mM MgCl_2_, 80 mM KCl, 0.5 mM EGTA, 10% glycerol, 10 mM β-glycerophosphate, 1 mM DTT, 0.05% NP40), 10 µl of an energy-regenerating-system (300 mM creatine phosphate, 10 mg/ml creatine kinase, 30 mM MgCl_2_, 10 mM DTT, 30 mM ATP pH 8), to reach a total volume of 100 µl. After 6 to 8 hours of assembly at 26°C, chromatin was digested for 1 min with MNase at 2–60 U/ml concentrations. Reactions were stopped by adding one volume of TEES/proteinase K (10 mM Tris HCl pH 7.5, 10 mM EDTA, 10 mM EGTA, 1% SDS, 50 µg/ml proteinase K) and incubated at 37°C for 2 hours. DNA was phenol-extracted and run on a 1.5% agarose gel.

## Supporting Information

Figure S1 **TRF2 alters nucleosomal organization in HT1080 fibrosarcoma cells.** (**A**) ChIP of HT1080 cells overexpressing TRF2^FL^ or TRF2^ΔBΔM^ and of control HT1080 cells using the indicated antibodies. Slot-blots were hybridized with a labelled Telo repeat probe and an Alu probe. (**B**) Quantification of the data in (**A**) expressed as probe/input hybridization signals. Error bars are s.d. of three independent experiments. Asterisks, p<0.05 based on unpaired Student's t-test.
(TIF)

Figure S2 **The influence of TRF2 on nucleosome organization does not derive from telomere shortening.** (**A**) Terminal restriction fragment length measured by Southern Blot in C33A cells infected with an empty vector, with TRF2^FL^ or TRF2^ΔBΔM^ hybridized with the telomeric probe (TTAGGG)_4_. (**B**) Quantification of the data in (A) expressed as mean telomere length. (**C**) Terminal restriction fragment length measured by Southern Blot in HT1080 cells infected with an empty vector, with TRF2^FL^ or TRF2^ΔBΔM^ hybridized with the telomeric probe (TTAGGG)_4_. (**D**) Quantification of the data in (C) expressed as mean telomere length.
(TIF)

Figure S3 **Chromatin assembly on non-repetitive DNA sequence.** MNase digestion of chromatin assembled on the linearized pUC18/601 plasmid. Lane 1, labelled 100 bp DNA ladder; lane 2, assembled chromatin digested with 60 U/ml of MNase. A schematic drawing of the DNA fragment and of the nucleosomal positioning and spacing is represented on the right.
(TIF)

Figure S4 **Non-specific proteins do not alter nucleosome spacing at telomeres in in vitro chromatin assembly.** MNase digestion of chromatin assembled on the 601/telomere DNA fragment. Lane 1, labelled 100 bp DNA ladder; lane 2, assembled chromatin digested with 60 U/ml of MNase; lane 3, assembled chromatin digested with 60 U/ml of MNase in the presence of 200 nM BSA.
(TIF)

Figure S5 **Gel analysis of recombinant TRF2 protein.** SDS-page gel analysis of the TRF2 protein after purification. Lane 1, protein ladder; lane 2–3, TRF2 protein, 0.5 µg and 4 µg respectively.
(TIF)
