# Supplementary figures and images for: TRF2 Controls Telomeric Nucleosome Organization in a Cell Cycle Phase-Dependent Manner
Source: PLoS One. 2012 Apr 20;7(4):e34386. doi: 10.1371/journal.pone.0034386 (PMC3335031; doi:10.1371/journal.pone.0034386)

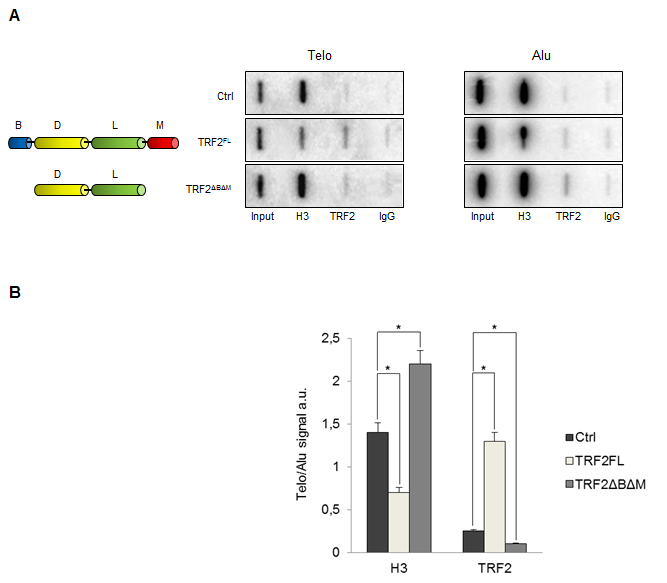

Supplement: Figure S1 — TRF2 alters nucleosomal organization in HT1080 fibrosarcoma cells. (A) ChIP of HT1080 cells overexpressing TRF2FL or TRF2ΔBΔM and of control HT1080 cells using the indicated antibodies. Slot-blots were hybridized with a labelled Telo repeat probe and an Alu probe. (B) Quantification of the data in (A) expressed as probe/input hybridization signals. Error bars are s.d. of three independent experiments. Asterisks, p<0.05 based on unpaired Student's t-test. (TIF) [file pone.0034386.s001.tif]

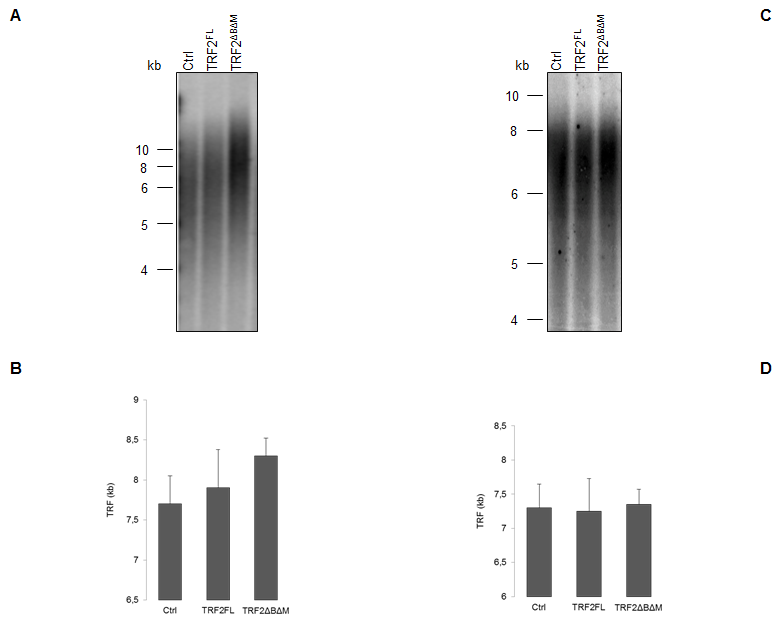

Supplement: Figure S2 — The influence of TRF2 on nucleosome organization does not derive from telomere shortening. (A) Terminal restriction fragment length measured by Southern Blot in C33A cells infected with an empty vector, with TRF2FL or TRF2ΔBΔM hybridized with the telomeric probe (TTAGGG)4. (B) Quantification of the data in (A) expressed as mean telomere length. (C) Terminal restriction fragment length measured by Southern Blot in HT1080 cells infected with an empty vector, with TRF2FL or TRF2ΔBΔM hybridized with the telomeric probe (TTAGGG)4. (D) Quantification of the data in (C) expressed as mean telomere length. (TIF) [file pone.0034386.s002.tif]

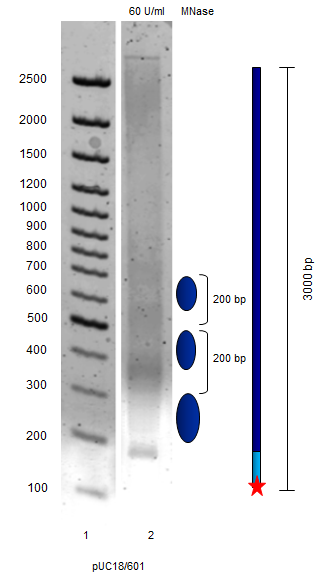

Supplement: Figure S3 — Chromatin assembly on non-repetitive DNA sequence. MNase digestion of chromatin assembled on the linearized pUC18/601 plasmid. Lane 1, labelled 100 bp DNA ladder; lane 2, assembled chromatin digested with 60 U/ml of MNase. A schematic drawing of the DNA fragment and of the nucleosomal positioning and spacing is represented on the right. (TIF) [file pone.0034386.s003.tif]

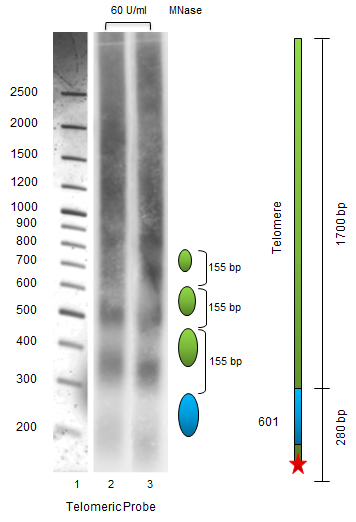

Supplement: Figure S4 — Non-specific proteins do not alter nucleosome spacing at telomeres in in vitro chromatin assembly. MNase digestion of chromatin assembled on the 601/telomere DNA fragment. Lane 1, labelled 100 bp DNA ladder; lane 2, assembled chromatin digested with 60 U/ml of MNase; lane 3, assembled chromatin digested with 60 U/ml of MNase in the presence of 200 nM BSA. (TIF) [file pone.0034386.s004.tif]

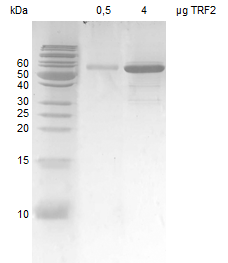

Supplement: Figure S5 — Gel analysis of recombinant TRF2 protein. SDS-page gel analysis of the TRF2 protein after purification. Lane 1, protein ladder; lane 2–3, TRF2 protein, 0.5 µg and 4 µg respectively. (TIF) [file pone.0034386.s005.tif]
